# Development of a Blueprint for Integrated Care for Vulnerable Pregnant Women

**DOI:** 10.1007/s10995-021-03340-y

**Published:** 2022-01-09

**Authors:** H. W. Harmsen van der Vliet-Torij, A. A. Venekamp, H. J. M. van Heijningen-Tousain, E. Wingelaar-Loomans, J. Scheele, J. P. de Graaf, M. P. Lambregtse-van den Berg, E. A. P. Steegers, M. J. B. M. Goumans

**Affiliations:** 1 Research Center Innovations in Care, Rotterdam University of Applied Sciences, Rochussenstraat 198, 3015 EK Rotterdam, The Netherlands; 2 Department of Obstetrics and Gynaecology, Erasmus MC, Room Wk-221, PO Box 2040, 3000 CA Rotterdam, The Netherlands; 3 Department of Child and Adolescent Psychiatry, Erasmus MC, PO Box 2040, 3000 CA Rotterdam, The Netherlands; 4 Department of Psychiatrie, Erasmus MC, PO Box 2040, 3000 CA Rotterdam, The Netherlands

**Keywords:** Perinatal care, Vulnerability, Practice-based research, Integrated care, Blueprint

## Abstract

**Purpose:**

There has been increasing awareness of perinatal health and organisation of maternal and child health care in the Netherlands as a result of poor perinatal outcomes. Vulnerable women have a higher risk of these poor perinatal outcomes and also have a higher chance of receiving less adequate care. Therefore, within a consortium, embracing 100 organisations among professionals, educators, researchers, and policymakers, a joint aim was defined to support maternal and child health care professionals and social care professionals in providing adequate, integrated care for vulnerable pregnant women.

**Description:**

Within the consortium, vulnerability is defined as the presence of psychopathology, psychosocial problems, and/or substance use, combined with a lack of individual and/or social resources. Three studies focussing on population characteristics, organisation of care and knowledge, skills, and attitudes of professionals regarding vulnerable pregnant women, were carried out. Outcomes were discussed in three field consultations.

**Assessment:**

The outcomes of the studies, followed by the field consultations, resulted in a blueprint that was subsequently adapted to local operational care pathways in seven obstetric collaborations (organisational structures that consist of obstetricians of a single hospital and collaborating midwifery practices) and their collaborative partners. We conducted 12 interviews to evaluate the adaptation of the blueprint to local operational care pathways and its’ embedding into the obstetric collaborations.

**Conclusion:**

Practice-based research resulted in a blueprint tailored to the needs of maternal and child health care professionals and social care professionals and providing structure and uniformity to integrated care provision for vulnerable pregnant women.

## Significance

Vulnerable women have a higher risk of perinatal morbidity and mortality. Unfortunately, these women also have a higher chance of receiving less adequate care.

The process that is described in this article might be applicable to other countries and communities to develop integrated care for vulnerable pregnant women and to adapt this integrated care to local communities. The blueprint might be applicable as a framework to ascertain arrangements for vulnerable pregnant women between maternal and child health care professionals and social care professionals, and to include these arrangements within routine antenatal care within communities.

## Introduction

There has been increasing awareness of perinatal health and the organisation of maternal and child health care in the Netherlands, because Dutch perinatal outcomes, with a perinatal mortality rate of 7.1 per 1000 in 2004, 5.1 per 1000 in 2010, and 4.2 per 1000 in 2015, appeared to be worse than in most other European countries (Bonsel et al., 2010; Buitendijk et al., 2003; de Jonge et al., 2009; EURO-PERISTAT, 2008, 2010, 2015). Vulnerable women have a higher risk of worse perinatal outcomes such as severe perinatal morbidity and perinatal mortality. These outcomes are particularly present in deprived neighbourhoods, where many of these vulnerable women live (De Graaf et al., 2013; Poeran et al., 2011; Posthumus, 2016). Differences in perinatal outcomes between neighbourhoods can be explained by individual medical risk factors among pregnant women and by differences in psychosocial, non-medical risk factors, such as a low socioeconomic status, a weak social cohesion and unsafe neighbourhoods (Posthumus, 2016; Timmermans et al., 2011). In addition, vulnerable pregnant women have a higher chance of receiving less adequate care due to the complexity of case management as they often face an accumulation of risk factors (Posthumus, 2016). Therefore, it is important that both maternal and child health care professionals and social care professionals take both medical and non-medical risk factors relevant to a pregnant woman and her social and physical environment into account. However, the integration of both maternal and child health care and social care is not part of routine antenatal care in the Netherlands.

As a response to poorer perinatal outcomes in the Netherlands, a large consortium embracing 100 organisations among professionals, educators, researchers and policymakers was set up in the Southwest region of the Netherlands. This consortium has set a joint aim to contribute to the quality of maternal and child health care and to improve integrated care between professionals within and between communities and clinical settings, by conducting practice-based research. Also, the consortium has established a joint research focus on vulnerable pregnant women. Within the consortium, vulnerability is defined as the combined presence of psychopathology (past and present), psychosocial problems, and/or substance use, combined with a lack of individual and/or social resources such as low education or being health illiterate (De Groot et al., 2016). Therefore, within the consortium, to support maternal and child health care professionals and social care professionals in providing adequate, integrated care for vulnerable pregnant women, we developed a blueprint.

In this article, the development process towards a blueprint, the blueprint itself that can serve as a framework, and its’ adaptation to local operational care pathways, is described.

## Description

Based on challenges from maternal and child health practice, indicated by professionals, three studies were conducted that focused on (a) population characteristics of pregnant women, (b) organisation of maternal and childbirth care concerning vulnerable pregnant women, and (c) knowledge, skills and attitudes of professionals about vulnerable pregnant women in the Southwest region of the Netherlands. During these studies, we gained more insight into the problems that professionals encountered in daily practice. Professionals mentioned that available care appeared to be fragmented. One of them told us:

…. there is rather too much than too little care provision…. Everything works isolated and you need to fit in a box in order to be helped.

Another professional said:

In particular, the guidance to specific care is difficult, because then other organizations need to be contacted and that is difficult. It is hard to approach the right person…. A doesn’t know from B who does what and knowledge is not always present.

Also, identifying women with psychosocial problems was a focus for birthcare professionals. Someone told us:

Recently, during a home visit, it became clear that the client was a vulnerable pregnant woman. In collaboration with the maternity care organisation we identified this lady as a vulnerable pregnant woman. She had financial problems, children under supervision of youth care. Although we did ask for this during consultations, we didn’t notice.

**Fig. 1:**
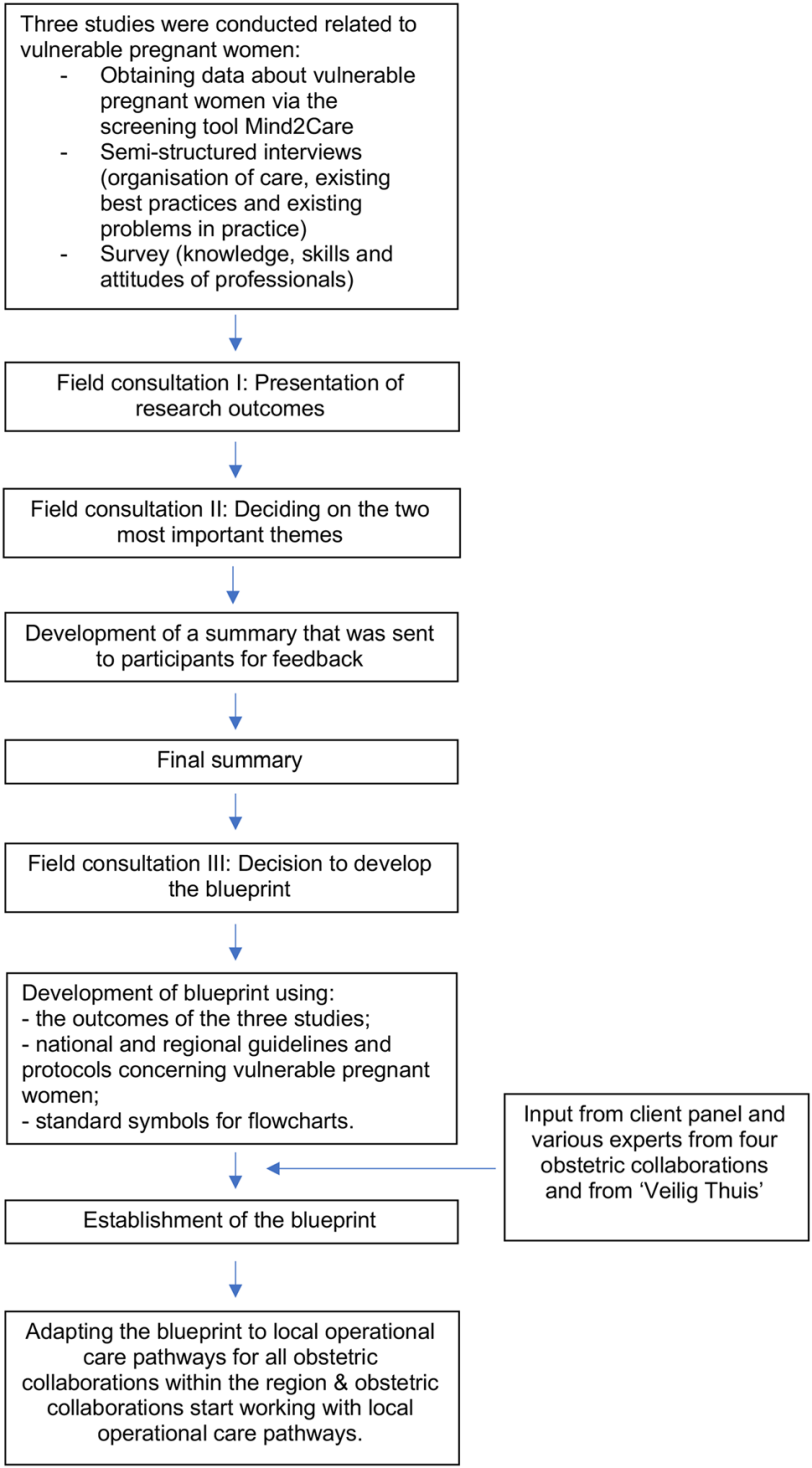
Development process of the blueprint

Outcomes were discussed with participants of the consortium in three field consultations. During the first field consultation, as shown in Fig. 1, participants were asked whether they recognised the studies’ outcomes, could agree with the studies’ outcomes and whether they had additional comments. In the second field consultation, best practices and gaps in knowledge, skills and/or tools in practice that emerged from the studies, were printed on large sheets of paper. Participants were divided into four groups and each group consisted of 20–25 participants. Within these groups, participants were asked to choose two themes that were most important to them by placing green stickers at these themes indicated on the sheets of paper and two themes that were of least importance by placing two red stickers. Subsequently, participants discussed the reasons for their choices. The four groups unanimously indicated two themes as their focal points: (1) to structure care specifically for pregnant women suffering from psychosocial problems (e.g. lack of social support, relational problems, financial problems, housing problems) and (2) to improve cooperation between maternal and child health care professionals and the organisation ‘Veilig Thuis’ (‘Safe at Home’), a national organisation that supports families that face domestic violence and/or child abuse.

Outcomes of this second field consultation, were summarized and then sent to the advisory board of the consortium and to a minimum of two participants from each of the four groups in the second field consultation. All professionals who received this summary, responded and their feedback was processed in a final summary. During this process the idea arose to develop a blueprint as a framework in which the results of the three studies and the field consultations could be embedded. The final summary and the idea of developing a blueprint was adopted during the third field consultation, which was the starting point for the development of the blueprint.

After that, the blueprint was developed using (a) the outcomes of the studies and the field consultations, (b) national (Augeo, 2013) and regional guidelines and protocols relating to vulnerable pregnant women and (c) standard symbols for flowcharts. During the development of the blueprint we closely collaborated with professionals from four obstetric collaborations—organisational structures that consist of the obstetricians of a single hospital and their collaborating midwifery practices who refer women to this hospital (Posthumus, 2016), professionals from Veilig Thuis (‘Safe at Home’), and social care professionals, who read drafts of the blueprint and provided input and advice. Furthermore, the client panel related to the consortium, that consists of 5–7 (future) mothers with diverse backgrounds and that advises the consortium about the benefits of the studies for pregnant women, was asked for input and advice.

**Fig. 2:**
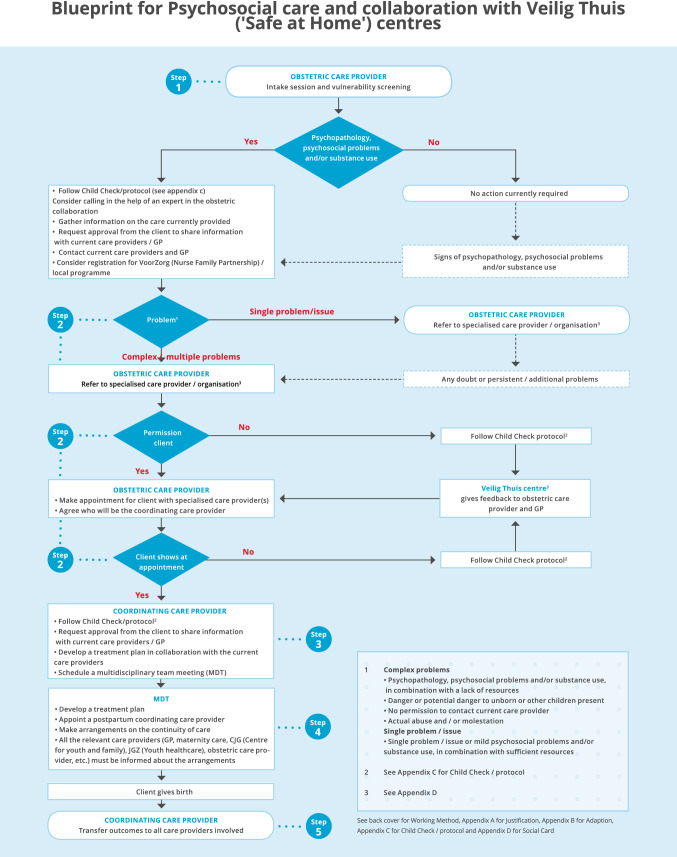

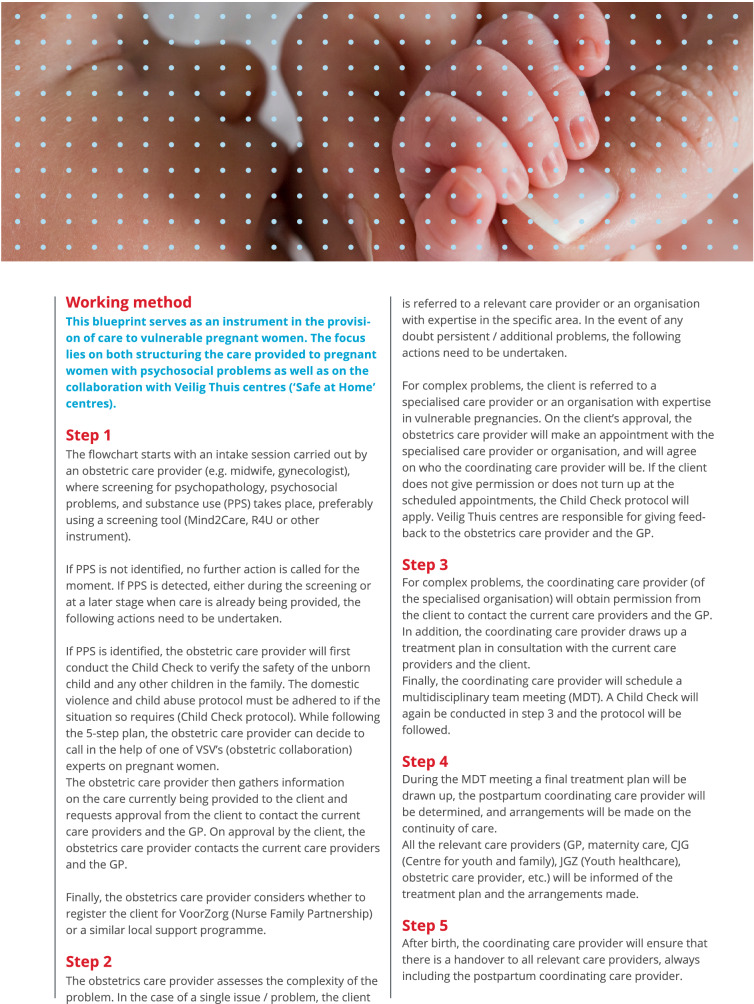
Blueprint for Psychosocial care and collaboration with Veilig Thuis (‘Safe at Home’) centres. Appendix A contains a justification for the blueprint for practice; Appendix C contains a flowchart developed by Augeo (2013), a national organisation that supports families that face domestic violence and/or child abuse; Appendix D is empty and can be filled by the obstetric collaborations themselves

The development process of the blueprint is visualised in Fig. 1. The blueprint itself is shown in Fig. 2.

## Assessment

**Fig. 3:**
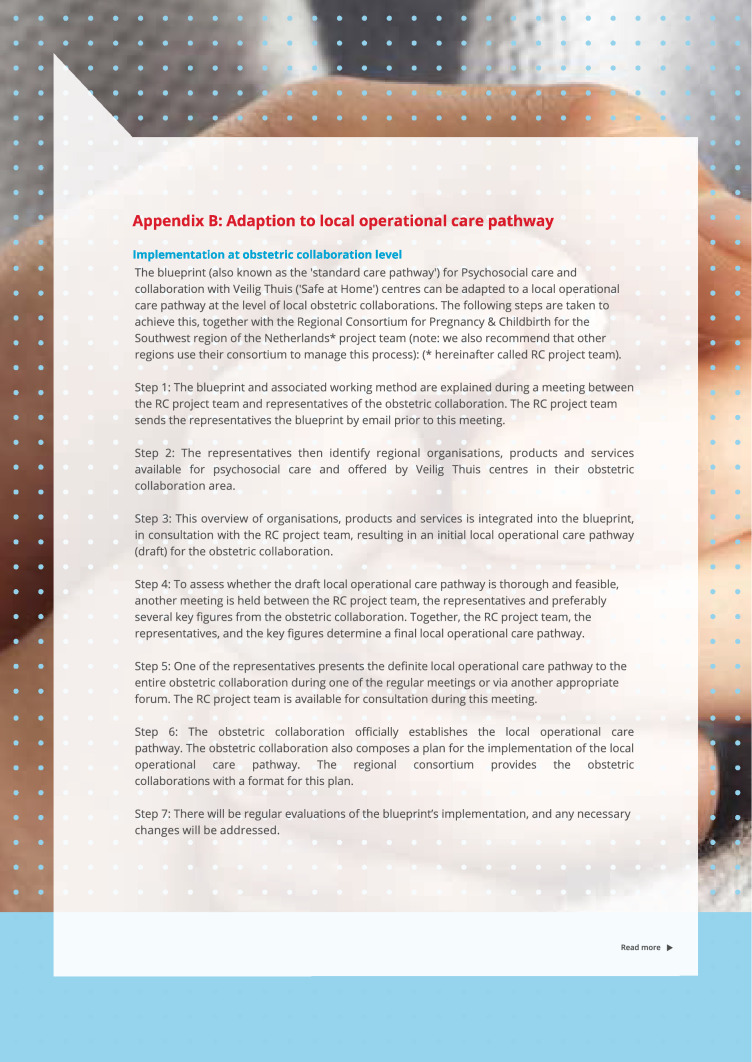

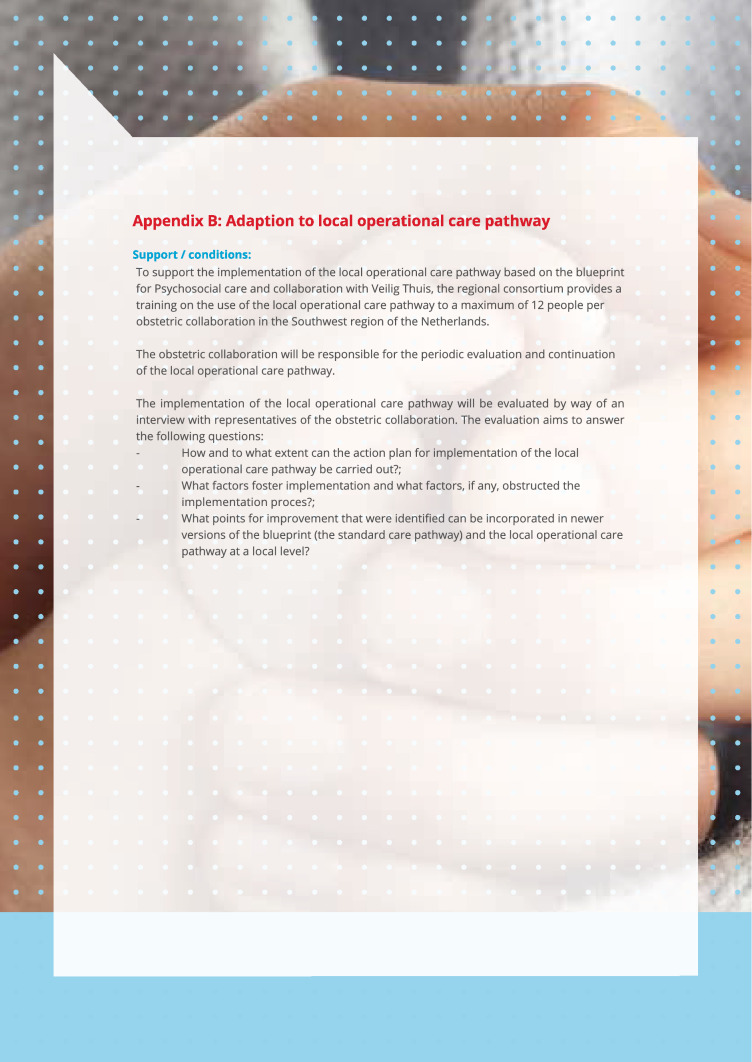
Adaptation of the blueprint to local operational care pathways

The blueprint has been developed as a framework for integrated care provision for vulnerable pregnant women. After its development, the blueprint was adapted to seven local operational care pathways for obstetric collaborations, in collaboration with members of the obstetric collaborations. This adaptation was a first step towards embedding of integrated care provision for vulnerable pregnant women on a local level. To adapt the blueprint to local operational care pathways, seven steps were followed, as shown in attachment B in Fig. 3: (a) The blueprint and the process of tailoring the blueprint to local circumstances for each obstetric collaboration were explained during a meeting between the project team of the consortium and representatives of the obstetric collaboration; (b) These representatives then identified regional organisations and services available for psychosocial care and offered by Veilig Thuis (‘Safe at Home’) centres in their community; (c) The overview of these products and services was incorporated into the blueprint, resulting in an initial local operational care pathway (draft) for the obstetric collaboration; (d) To assess whether the draft was thorough and feasible, another meeting was held between the project team, the representatives and, where possible, several key figures from the obstetric collaboration to determine a final care pathway; (e) One of the representatives presented the final local operational care pathway to the entire obstetric collaboration during one of the regular meetings or via another appropriate forum. The project team was present for consultation during this meeting; (f) The obstetric collaboration established the local operational care pathway and composed a plan for implementation. The consortium provided the obstetric collaborations with a format for this plan; (g) Regular evaluations of the blueprint’s implementation process were committed by the RC project team and any necessary changes were addressed.

Several professionals, representing seven obstetric collaborations, followed a multidisciplinary training organized by the consortium to contribute to successful embedding of the local operational care pathways.

We conducted 12 semi-structured interviews to evaluate the adaptation of the blueprint to local operational care pathways and its’ embedding into the obstetric collaborations. The interviewees represented all nine obstetric collaborations within the region, seven of which successfully adapted the blueprint to a local operational care pathway and started working with it. In two of the nine obstetric collaborations, adaptation to local operational care pathways failed due to lack of resources to support adaptation. One of the obstetric collaborations in which adaptation was not successful did use the draft of their blueprint as fundament for discussion about the need for resources to improve care for vulnerable pregnant women in their hospital.

Almost all interviewees experienced the *adaption of the blueprint towards local operational care pathways* as a comprehensive approach. Although, there were large differences in the division of tasks between obstetric collaborations during the adaptation process. In one obstetric collaboration several working groups were set up, but in a few others the responsibility of the adaption rested on one or two people. Due to workload or inadequate budget for extra working hours, adaptation took a long time in some obstetric collaborations. Almost all interviewees mentioned that the adaptation towards local obstetric care pathways took a lot of effort and time. Despite these struggles, all interviewees valued the adaptation to local operational care pathways as positive. Main reasons mentioned were that care for vulnerable pregnant women was now organised and structured in the same way and there was less variation between care providers as everyone used the same pathway. Half of the interviewees mentioned that they wanted to involve local government in further development of the local operational care pathway. A few obstetric collaborations already started cooperation with local government. Almost all interviewees mentioned that *working with the local operational care pathway* provided structure in their work with vulnerable pregnant women. They used it as reference work, also when they had less experience with providing care for vulnerable pregnant women. However, some interviewees were worried that in the future professionals would forget to use it, due to stress and busy agendas (Wingelaar-Loomans et al., not published).

## Conclusion

### Discussion

Our aim was to support maternal and child health care professionals and social care professionals in providing adequate, integrated care for vulnerable pregnant women. A joint process resulted in a blueprint for integrated care provision for vulnerable pregnant women that was successfully adapted to local operational care pathways.

Literature describes the importance of continuity of care and integrated care, but hardly touches upon the issue of integration of both maternal and child health care and social care as part of routine antenatal care. De Groot et al. (2016) described the importance of structured care for vulnerable pregnant women for professionals as units (midwifery practice, obstetric unit) with a high degree of structured care show lower burden in workload compared to units with the same objective caseload of vulnerable clients. This outcome suggests that structured care contributes to a decrease in workload of professionals. Dawson et al. (2015) emphasized the importance of a conceptual framework to support (and scale up) nurses’ and midwives’ roles in improving ‘access’ to primary health care for vulnerable populations. Also, literature about specific risk factors (e.g. smoking, use of alcohol), existing policies and the role of professionals can be found (Bartholomew & Abouk, 2016; Flemming et al., 2016; Havard et al., 2018; Milligan et al., 2010; O'Leary & Bower, 2012; Roberts et al., 2017; Vicedo-Cabrera et al., 2016). However, literature that describes frameworks that combine these risk factors and integrated care for vulnerable pregnant women between maternal and child health and social care professionals as we developed and integrated in the blueprint, is nonexistent. Only blueprints or protocols for integrated care for specific risk factors are present, as Kilbourne et al. (2002) described for depression.

One of the strengths of the process of developing the blueprint is that maternal and child health and social care professionals were involved in all phases of the studies that were carried out. Also, they were involved in the field consultations and during the development of the blueprint. Various experts and clients of the consortiums’ client panel also provided input and advice. A weakness is that we probably included mainly professionals who are willing to improve care for vulnerable pregnant women and that we might not have succeeded in reaching out to all professionals, especially those professionals who are less motivated. The consortiums’ client panel consists of (future) mothers with diverse, including vulnerable, backgrounds. At the same time, they might not represent the opinion of all (future) mothers in the region. Culture of the mother and her family, for example, is an important distinguishing variable that can influence vulnerable women’s perinatal experiences and expectations. Investigating this variable and exploring and discussing the influence of culture together with the pregnant woman in an explicit and candid way is important (Barkensjö et al., 2018; Son, 2016; Watson et al., 2019). Although the process described in this article focusses on the development of a tool that supports professionals, adequate care provision also includes that maternal and child health care professionals investigate expectations and experiences of women and that they discuss these expectations and experiences with women themselves.

We evaluated the blueprint and the adaptation process through interviews. A weakness of our process might be that the outcomes of the interviews possibly do not represent the opinions of all members of the obstetric collaborations. Obstetric collaborations started working with the local operational care pathways, which is a successful first step. We consulted an implementation expert during the development of the blueprint, but there was no structural involvement of an implementation scientist. The continuous involvement of an implementation scientist is needed to ensure successful implementation of the local operational care pathways in future. As described by Bauer et al. (2015) evidence-based implementation strategies are key in ensuring that research investments contribute to improving healthcare value and public health, and implementation science plays an important role in supporting these efforts.

### Conclusion for Practice

The blueprint is a framework for integrated care provision for vulnerable pregnant women and is feasible and well accepted for the region. Working with local operational care pathways may have contributed to better cooperation between professionals and to uniformity in the integrated care provision for vulnerable pregnant women. We expect that the blueprint could be adopted by and tailored to local circumstances of other obstetric collaborations in the Netherlands. At least two obstetric collaborations outside the region, are interested in adapting the blueprint to their local care practice. International use of the blueprint can be a challenge due to differences in organisation of healthcare, different populations and cultural differences. However, other countries could follow the same approach as we described to develop tools for integrated care and to apply these developed tools to their organisation of health care. As far as we know, the blueprint and the preceding approach to develop the blueprint is unique, and integration of the maternal and child health care and social care domain as part of routine antenatal care is unusual.

More research is needed to find out whether integration of the maternal and child health care and social care domain as part of routine antenatal care leads to better quality of care and improved perinatal outcomes among vulnerable pregnant women.
